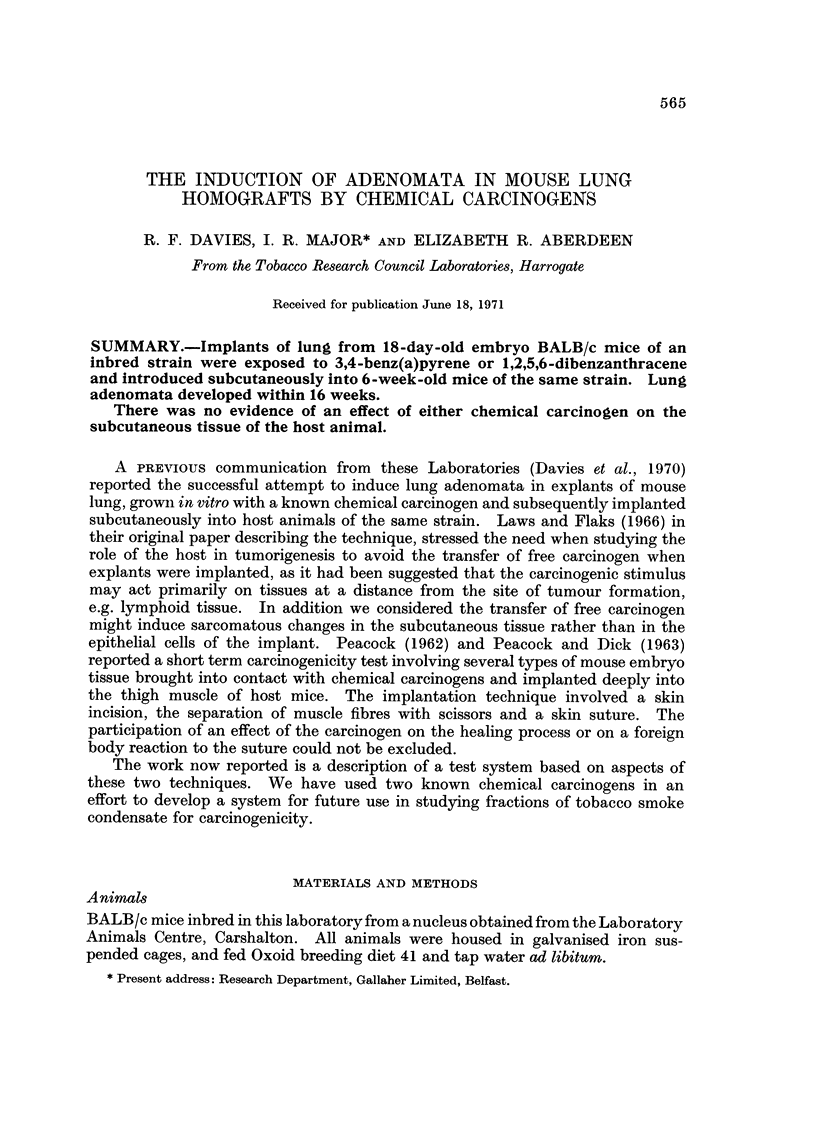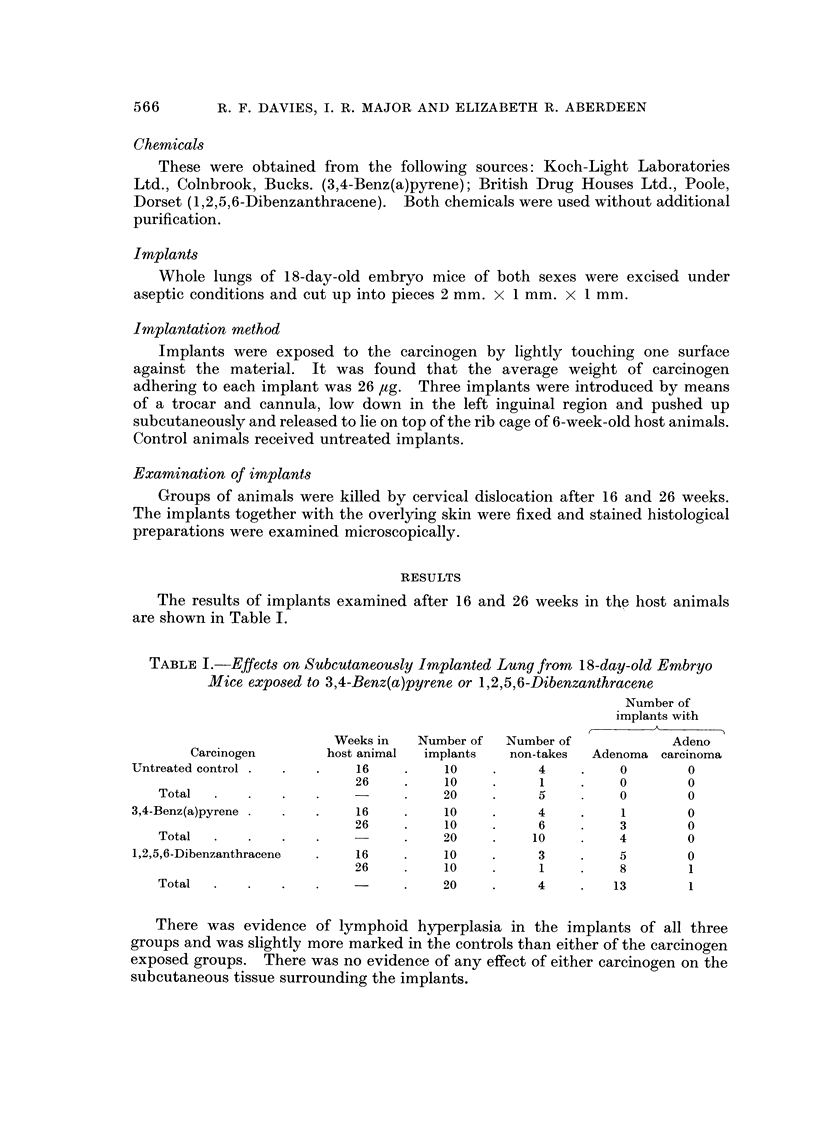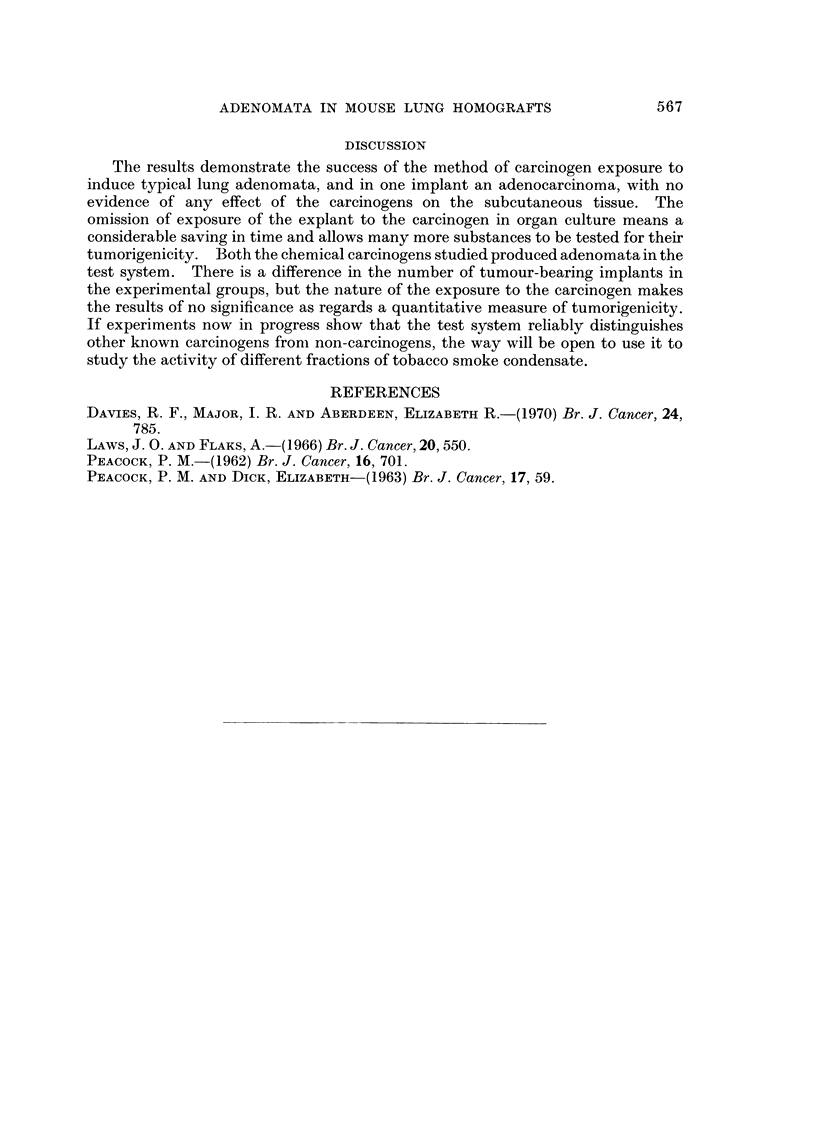# The Induction of Adenomata in Mouse Lung Homografts by Chemical Carcinogens

**DOI:** 10.1038/bjc.1971.71

**Published:** 1971-09

**Authors:** R. F. Davies, I. R. Major, Elizabeth R. Aberdeen

## Abstract

Implants of lung from 18-day-old embryo BALB/c mice of an inbred strain were exposed to 3,4-benz(a)pyrene or 1,2,5,6-dibenzanthracene and introduced subcutaneously into 6-week-old mice of the same strain. Lung adenomata developed within 16 weeks.

There was no evidence of an effect of either chemical carcinogen on the subcutaneous tissue of the host animal.


### THE INDUCTION OF ADENOMATA IN MOUSE LUNG HOMOGRAFTS BY CHEMICAL CARCINOGENS

R. F. DAVIES, I. R. MAJOR* AND ELIZABETH R. ABERDEEN

*From the Tobacco Research Council Laboratories, Harrogate*

Received for publication June 18, 1971

**SUMMARY.—Implants of lung from 18-day-old embryo BALB/c mice of an inbred strain were exposed to 3,4-benz(a)pyrene or 1,2,5,6-dibenzanthracene and introduced subcutaneously into 6-week-old mice of the same strain. Lung adenomata developed within 16 weeks.**

**There was no evidence of an effect of either chemical carcinogen on the subcutaneous tissue of the host animal.**

A PREVIOUS communication from these Laboratories (Davies *et al.*, 1970) reported the successful attempt to induce lung adenomata in explants of mouse lung, grown *in vitro* with a known chemical carcinogen and subsequently implanted subcutaneously into host animals of the same strain. Laws and Flaks (1966) in their original paper describing the technique, stressed the need when studying the role of the host in tumorigenesis to avoid the transfer of free carcinogen when explants were implanted, as it had been suggested that the carcinogenic stimulus may act primarily on tissues at a distance from the site of tumour formation, e.g. lymphoid tissue. In addition we considered the transfer of free carcinogen might induce sarcomatous changes in the subcutaneous tissue rather than in the epithelial cells of the implant. Peacock (1962) and Peacock and Dick (1963) reported a short term carcinogenicity test involving several types of mouse embryo tissue brought into contact with chemical carcinogens and implanted deeply into the thigh muscle of host mice. The implantation technique involved a skin incision, the separation of muscle fibres with scissors and a skin suture. The participation of an effect of the carcinogen on the healing process or on a foreign body reaction to the suture could not be excluded.

The work now reported is a description of a test system based on aspects of these two techniques. We have used two known chemical carcinogens in an effort to develop a system for future use in studying fractions of tobacco smoke condensate for carcinogenicity.

### MATERIALS AND METHODS

### *Animals*

BALB/c mice inbred in this laboratory from a nucleus obtained from the Laboratory Animals Centre, Carshalton. All animals were housed in galvanised iron suspended cages, and fed Oxoid breeding diet 41 and tap water *ad libitum*.

* Present address: Research Department, Gallaher Limited, Belfast.

*Chemicals*

These were obtained from the following sources: Koch-Light Laboratories Ltd., Colnbrook, Bucks. (3,4-Benz(a)pyrene); British Drug Houses Ltd., Poole, Dorset (1,2,5,6-Dibenzanthracene). Both chemicals were used without additional purification.

*Implants*

Whole lungs of 18-day-old embryo mice of both sexes were excised under aseptic conditions and cut up into pieces 2 mm. × 1 mm. × 1 mm.

*Implantation method*

Implants were exposed to the carcinogen by lightly touching one surface against the material. It was found that the average weight of carcinogen adhering to each implant was 26 μg. Three implants were introduced by means of a trocar and cannula, low down in the left inguinal region and pushed up subcutaneously and released to lie on top of the rib cage of 6-week-old host animals. Control animals received untreated implants.

*Examination of implants*

Groups of animals were killed by cervical dislocation after 16 and 26 weeks. The implants together with the overlying skin were fixed and stained histological preparations were examined microscopically.

### RESULTS

The results of implants examined after 16 and 26 weeks in the host animals are shown in Table I.

TABLE I.—*Effects on Subcutaneously Implanted Lung from 18-day-old Embryo Mice exposed to 3,4-Benz(a)pyrene or 1,2,5,6-Dibenzanthracene*

| Carcinogen | Weeks in host animal | Number of implants | Number of non-takes | Number of implants with: Adenoma | Number of implants with: Adeno carcinoma |
|---|---|---|---|---|---|
| Untreated control | 16 | 10 | 4 | 0 | 0 |
| | 26 | 10 | 1 | 0 | 0 |
| Total | — | 20 | 5 | 0 | 0 |
| 3,4-Benz(a)pyrene | 16 | 10 | 4 | 1 | 0 |
| | 26 | 10 | 6 | 3 | 0 |
| Total | — | 20 | 10 | 4 | 0 |
| 1,2,5,6-Dibenzanthracene | 16 | 10 | 3 | 5 | 0 |
| | 26 | 10 | 1 | 8 | 1 |
| Total | — | 20 | 4 | 13 | 1 |

There was evidence of lymphoid hyperplasia in the implants of all three groups and was slightly more marked in the controls than either of the carcinogen exposed groups. There was no evidence of any effect of either carcinogen on the subcutaneous tissue surrounding the implants.

### DISCUSSION

The results demonstrate the success of the method of carcinogen exposure to induce typical lung adenomata, and in one implant an adenocarcinoma, with no evidence of any effect of the carcinogens on the subcutaneous tissue. The omission of exposure of the explant to the carcinogen in organ culture means a considerable saving in time and allows many more substances to be tested for their tumorigenicity. Both the chemical carcinogens studied produced adenomata in the test system. There is a difference in the number of tumour-bearing implants in the experimental groups, but the nature of the exposure to the carcinogen makes the results of no significance as regards a quantitative measure of tumorigenicity. If experiments now in progress show that the test system reliably distinguishes other known carcinogens from non-carcinogens, the way will be open to use it to study the activity of different fractions of tobacco smoke condensate.

### REFERENCES

DAVIES, R. F., MAJOR, I. R. AND ABERDEEN, ELIZABETH R.—(1970) *Br. J. Cancer*, **24**, 785.

LAWS, J. O. AND FLAKS, A.—(1966) *Br. J. Cancer*, **20**, 550.

PEACOCK, P. M.—(1962) *Br. J. Cancer*, **16**, 701.

PEACOCK, P. M. AND DICK, ELIZABETH—(1963) *Br. J. Cancer*, **17**, 59.